# Endophytic Diversity in *Vitis vinifera* with Different Vineyard Managements and *Vitis sylvestris* Populations from Northern Italy: A Comparative Study of Culture-Dependent and Amplicon Sequencing Methods

**DOI:** 10.3390/biology14030293

**Published:** 2025-03-14

**Authors:** Simona Pizzi, Angela Conti, Alessandra Di Canito, Debora Casagrande Pierantoni, Roberto Foschino, Mathabatha Evodia Setati, Ileana Vigentini

**Affiliations:** 1 Department of Biomedical, Surgical and Dental Sciences (DISBIOC), University of Milan, 20122 Milan, Italy; simona.pizzi1@unimi.it (S.P.); alessandra.dicanito@unimi.it (A.D.C.); roberto.foschino@unimi.it (R.F.); 2 Department of Pharmaceutical Sciences (DSF), University of Perugia, 06123 Perugia, Italy; angela.conti@unipg.it (A.C.); debora.casagrandepierantoni@unipg.it (D.C.P.); 3 South African Grape and Wine Research Institute, Stellenbosch University, Matieland 7602, South Africa; setati@sun.ac.za

**Keywords:** grapevine endophytes, *Vitis vinifera*, *Vitis sylvestris*, metabarcoding, vineyard microbiome, biocontrol

## Abstract

This study aimed to investigate the endophytic microbial populations associated with wild and domesticated grapevines using both culture-based and culture-independent methods. Through culture-based methods, 148 endophytes were identified. The dominant fungal species included *Aureobasidium pullulans*, *Alternaria alternata*, and *Cladosporium allicinum*, while predominant bacterial species were *Ralstonia pikettii*, *Nocardia niigatensis*, and *Sphingomonas echinoides*. Culture-independent methods employed metagenomic techniques to explore microbial biodiversity, focusing on targeted amplification of bacterial 16S rRNA as well as fungal ITS and 26S rRNA gene regions. The main bacterial species identified included *Halomonas* sp., *Sphingomonas* sp. and *Massilia* sp., whereas the fungal population was dominated by *Cladosporium* sp., *Malassezia* sp. and *Mucor* sp. The findings revealed that vineyard management practices did not lead to statistically significant variations in microbial communities. The consistent presence of these genera across all samples suggests that they are stable components of the grapevine endophytic microbiota, remaining relatively unaffected by external environmental factors.

## Simple Summary

The environmental and human risks associated with the use of pesticides have increased the need for alternative approaches to disease control. Plants host a variety of microorganisms known as endophytes, which can confer benefits to their hosts. Exploring methods to isolate these organisms is crucial for evaluating their potential as biocontrol agents, with the ultimate goal of minimizing pesticide use in viticulture. This study investigated the endophytic microbial communities of two grapevine species: *Vitis vinifera*, cultivated under various vineyard management practices, and *Vitis sylvestris*, sampled from four distinct locations in Northern Italy.

## 1. Introduction

The agricultural industry relies on pesticides to enhance productivity and prevent crop diseases. However, excessive use poses significant risks to both the environment and human health. In viticulture, chemical products such as pesticides, herbicides, insecticides, fungicides, and copper are commonly applied to limit pathogen infections [1]. These residues can reach toxic levels, causing plant stress, reducing soil fertility, and affecting the safety of grapes and wine. To address these concerns, EC Directive No 128/2009 was introduced to promote the sustainable use of pesticides and minimize their application and environmental impact [2]. In September 2015, UN member countries worldwide signed the 2030 Agenda for Sustainable Development and its 17 Sustainable Development Goals (SDGs). Reducing chemical use aligns with several of these goals, particularly Goal 12 (Responsible Consumption and Production), as outlined in the “Farm to Fork” strategy [3].

In recent years, European farmers have expressed resistance to certain agricultural policies considered unfair, leading to protests and actions such as the withdrawal of pesticide-related legislation. These movements have also highlighted the need for increased farmer participation in policy -making. In response, the European Commission has prioritized non-chemical pest control and promoted the adoption of Integrated Pest Management (IPM) practices. From this point onward, IPM will be referred to as ‘conventional’ to facilitate comparisons with previous studies [4]. However, achieving a balance between environmental objectives and agricultural interests remains a complex and ongoing challenge.

Plants interact in the environment with a wide variety of microorganisms, including bacteria, filamentous fungi, and yeasts. These microorganisms can either benefit the host or act as pathogens. Some of them colonize the rhizosphere and phyllosphere, and can penetrate plant tissues, forming the endophytic population. Studies have demonstrated that endophytes colonize not only roots but also shoots, leaves, flowers, fruits, and reproductive organs of plants [5]. The beneficial effects directly associated with the metabolism of endophytes have garnered significant interest, particularly for their potential use as biological control agents (BCAs) to limit or prevent pathogen infections and, consequently, reduce pesticide use [6]. Some endophytes isolated from the grapevine counteract pathogen development through various mechanisms. For bacterial endophytes, the primary mechanisms for plant colonization and pathogen inhibition include antibiosis, production of lytic enzymes, δ-endotoxins, siderophores, and lipopeptides, as well as the induction of systemic resistance (ISR) in plants [7]. For fungal endophytes, additional mechanisms such as mycoparasitism and competition play a role [8]. Yeasts also exhibit unique strategies, such as tolerance to reactive oxygen species (ROS) and biofilm formation [9].

Endophyte populations in grapevines vary greatly depending on factors such as soil type, rootstock, plant age, and agricultural practices [10,11]. The comparison between integrated pest management (IPM) and organic production systems revealed differences in the bacterial and fungal endophytic communities in grapevines [10,12]. Furthermore, the distribution of endophytes varies within the different organs of the same plant, both qualitatively and quantitatively [13]. Several studies have reported a dominance of bacterial genera such as *Bacillus* spp. and *Staphylococcus* spp. [14,15] in leaves, as well as fungi like *Aspergillus* spp. and *Penicillium* spp. [16,17]. In grape berries, yeasts from genera *Metschnikowia* spp., *Pichia* spp., and *Hanseniaspora* spp., bacterial genera *Acinetobacter* spp., *Burkholderia* spp., and *Bacillus* spp., as well as fungi such as *Alternaria* spp. and *Cladosporium* spp., have been identified [18,19].

Plant-endophyte interactions hold significant potential for agriculture applications. However, the cultivation of endophytes under laboratory conditions remains challenging. Current methods for isolating cultivable endophytes involve surface sterilization to remove epiphytic microorganisms, followed by isolation and culturing on artificial media. Fluorescence microscopy techniques are used to detect endophytes within plant tissues, where their populations can vary due to migration from soil and movement through the plant’s vascular system [20]. Culture-independent methods also play a crucial role in evaluating grapevine microbial biodiversity. Techniques such as automated ribosomal intergenic spacer analysis (ARISA), denaturing gradient gel electrophoresis (DGGE), single-strand conformation polymorphism (SSCP), and metagenomic approaches based on the DNA sequencing of 16S rRNA and ITS1-5.8S-ITS2 rDNA regions are commonly used [21].

Despite recent advancements, a comprehensive understanding of grapevine-associated endophytic populations is still lacking. Comparative data from different geographical regions and cultivation systems could help define a stable core microbiota.

This study aimed to characterize the endophytic microbiota associated with wild and domesticated grapevines under different vineyard management systems (conventional, biological, biodynamic, and abandoned) using both culture-based and culture-independent methods.

## 2. Materials and Methods

### 2.1. Sampling

Grapevine material was collected from four locations in Northern Italy. For *V. vinifera*, six well-established vineyards were selected in Franciacorta (Lombardy) for each farming method: conventional, biological, and biodynamic. Samples from abandoned vineyards in Brescia (Lombardy, Italy) were collected. In Riccagioia (Lombardy, Italy), two Georgian cultivars resistant to *Plasmopara viticola* (the causal agent of downy mildew) were selected. These plants are part of the UMIL germplasm collection and were planted approximately twenty years ago under conventional management. For *V. vinifera* ssp. *sylvestris*, two populations were sampled: one in the Monte Fenera natural area (Piedmont), specifically in the “second” and “third” cores, and one in Montalto (Lombardy, Italy), approximately 100 m away from a cultivated field. Monte Fenera (VC) was the first location in Piedmont (Italy) where wild grapevines were identified. Sixteen grape plants, distributed across four different cores, were documented. The first core contained only two plants, while the fourth had one dead grapevine. The second and third cores are of particular interest due to their plant density and positioning. The second core is located on steep terrain with poor soil, which influences the arboreal composition, including species such as *Cornus mas* (cornelian cherry), *Fraxinus ornus* (manna ash), and hazel. The third core, situated on gentler slopes, supports species such as lime and cherry trees. Overall, wild grapevines were found exclusively in areas facing southwest, typically near vertical rock walls that retain heat and modify the microhabitat [22]. For the vineyards in Franciacorta and cultivated grapevines in Riccagioia, three healthy plants without visible disease symptoms were selected for microbiota and microbiome analysis. For wild grapevines, three dominant plants were sampled from each population. Details of each plot and the respective farming approach are presented in Table 1.

All the sampling took place between early May and mid-July 2022. To minimize variability across different areas, three plants were selected from each vineyard/population at the same phenological stage and, if possible, of similar size. These plants were spaced at least five metres apart and arranged in alternate rows. Lateral shoots were collected by cutting two portions, each 20–30 cm long, containing at least five leaves and one grape bunch. The samples were placed in sterile bags and transported to the laboratory in a portable refrigerator maintained at 4 °C.

In the laboratory, shoots, leaves, and grape berries were manually separated using sterile tools and subdivided according to the type of analysis. Samples designated for microbiota analysis were examined within 24 h of collection, while those intended for metabarcoding were externally sterilized and subsequently frozen at −80 °C.

### 2.2. Culture-Dependent Analysis

#### 2.2.1. Validation of the Sample’s Sterilization Step by Challenge Tests

To assess the efficacy of surface sterilization for endophyte isolation, a challenge test was performed by contaminating samples with a yeast strain of *Saccharomyces cerevisiae*. Shoots from a private vineyard and grape berries purchased from a supermarket were used. Experiments were performed in triplicate. To evaluate the initial surface contamination, three shoots (each 5 cm long) and three berries were treated with 30 mL of 0.1% (*v*/*v*) Tween 20 solution and then washed with sterile water for 5 min. A 10-mL aliquot of the wash water was transferred to a sterile tube and centrifuged at 1160× *g* 20 min (Hettich, ROTINA 380R, Tuttlingen, Germany). The pellet was resuspended in 100 µL of sterile water and the solution was spread onto APC medium (Avantor, Radnor, PA, USA). Plates were incubated at 25 °C for 14 days.

Depending on the material to be processed, two sterilization protocols (P) were applied. P1 was used for shoots, involving immersion in 90% ethanol for 3 min, followed by treatment with a 2.5% sodium hypochlorite solution for 3 min, and a final rinse with 30 mL of sterile water for 3 min. For berries, sterilization was performed using either the same protocol as for shoots (P1) or an alternative method (P2), which involved soaking them in 70% ethanol for 5 min, followed by a rinse with 30 mL of sterile water for 5 min.

For both protocols, the final 30 mL of wash water was tested as follows: 10 mL was centrifuged at 1160× *g* 20 min, the pellet was resuspended in 100 µL of sterile water and spread on APC medium (Sharlau, Barcelona, Spain); 10 mL was filtered by a sterile 0.22 µm filter (Millipore filter, type GSWP, Billerica, MA, USA) and the filter was placed onto APC medium; and 10 mL was enrichened in broth with APC composition without agar (*w*/*v* 0.25% yeast extract, *w*/*v* 0.5% tryptone, *w*/*v* 0.1% glucose). All tests were incubated at 25 °C for two weeks.

Preliminary sterilization was performed to obtain a sterile sample that could be subsequently contaminated.

Therefore, contamination with *S. cerevisiae* was conducted by immersing the samples in 30 mL of a cell suspension at 10^8^ cell/mL for 10 min, followed by drying for 4 h at 25 °C in a sterile environment. After contamination, the sterilization procedures were repeated, and the wash waters were tested as previously described.

#### 2.2.2. Endophyte Isolation

Endophytic populations were isolated from different parts of the plant organs (shoots, leaves and grape berries) from three different grapevine plants for each vineyard/population and analyzed within 48 h of sample collection. Samples underwent a surface sterilization process involving soaking in 90% ethanol for 3 min, followed by immersion in a 2.5% sodium hypochlorite solution for 3 min, and a final rinse with sterile water for 3 min. The effectiveness of the surface sterilization method was confirmed as previously described. After sterilization, different plant parts were processed using specific methods to optimize endophyte recovery. Shoots were sectioned into 0.5 cm pieces and placed on Potato Dextrose Agar (PDA) (Sharlau, Barcelona, Spain), APC medium with cycloheximide (100 mg/L) (Sharlau, Barcelona, Spain), and Yeast Glucose Chloramphenicol (YGC) (Sharlau, Barcelona, Spain) agar. The cut sections were oriented with the vascular vessels facing the medium and incubated for 7–15 days at 25 °C. Different media were used to evaluate the presence of various microbes. Leaves were cut into 1 cm × 1 cm portions and homogenized for 1 min at maximum speed using Minilys Personal Homogenizer (Bertin Technologies, Montigny-le-Bretonneux, France). The homogenate was plated on PDA medium and incubated under the same conditions as the shoots. Five berries from each sample were homogenized in 100 mL of 0.8% (*w*/*v*) NaCl solution, plated on Yeast Extract Peptone Dextrose (YEPD) medium with agar (*w*/*v* 1% yeast extract, *w*/*v* 2% meat peptone, *w*/*v* 2% glucose, *w*/*v* 2% agar), and incubated for 7–15 days at 25 °C. Following incubation, colonies with different morphologies that emerged from each plate were isolated onto PDA and stored in a 50% glycerol solution at −80 °C. Each sample was labelled with the prefix ED, followed by a progressive identification number.

#### 2.2.3. Fungal Identification

A preliminary classification of fungal isolates was conducted based on observation of colony shape, size, and color, followed by microscopic examination of cell morphology. This allowed for the separation of isolates into potential subpopulation, which were then analysed using molecular methods.

Fungal DNA extraction was conducted using a modified version of the protocol by Querol et al. (1992) [23]. Five mL of inoculum was prepared and incubated for 4 days at 25 °C. Then, the culture was centrifuged at 1160× *g* 20 min (Hettich, ROTINA 380R, Tuttlingen, Germany) and the pellet was treated according to Vigentini et al. (2012) [24].

The ITS regions of all fungal isolates were amplified using the primers ITS1 (5′-TCGGTAGGTGAACCT-3′) and ITS4 (5′-TCCTCCGCTTATTGA-3′) (Eurofins Genomics, Ebersberg, Germany) using a Mastercycler nexus (Eppendorf, Hamburg, Germany). For each 50 µL reaction, 80–100 ng of fungal gDNA was combined with PCR reaction buffer [1X] (GenScript, Piscataway, NJ, USA), MgCl_2_ [1 mM], deoxynucleotide triphosphate (dNTP) mixture [0.2 µM], two 15-base oligonucleotide primers [0.5 µM], Taq polymerase (2 U) (GenScript, Piscataway, NJ, USA) and molecular grade water. The PCR conditions were as follows: initial denaturation at 94 °C for 3 min, followed by 35 cycles of 94 °C for 1 min, 55 °C for 45 s, and 72 °C for 1 min, with a final extension at 72 °C for 5 min. Negative and positive controls were included to ensure the accuracy and validity of the results. The PCR products were visualized through gel electrophoresis (0.8% agarose, 0.4 mg/mL ethidium bromide, 0.5X TBE buffer) for 45 min at 100 V and 400 mA. DNA bands were visualised under UV light exposure and images were digitally acquired (Biorad, Hercules, CA, USA).

To generate clusters of isolates potentially belonging to the same fungal species, restriction fragment length polymorphism analysis of the ITS sequences (RFLP-ITS) was performed. In general, a fungal subpopulation was confirmed when all its members exhibited the same RFLP-ITS profile and shared corresponding morphological characteristics. For a 15 µL volume reaction, 10 µL of amplified DNA was mixed with 1.5 µL of Buffer Tango [10X], 0.5 µL of Hin6I (HinP1I) [10 U/µL] (Difco™, ThermoFischer Scientific, Waltham, MA, USA) and 3 µL of milliQ water. The reaction mixtures were incubated at 37 °C for 2 h, and the restriction profiles were analyzed on a 2.5% agarose gel containing 0.4 mg/mL ethidium bromide in 0.5X TBE buffer at 75 V and 400 mA for 2 h, alongside a 100 bp DNA marker (LeGene 100 bp DNA Ladder, Dye-mixed, 100 lanes). To attain species identification, ITS sequencing was performed on approximately 10% of the isolates in each subpopulation. Any unique RFLP profile that did not belong to other clusters was treated as an individual population and subjected to ITS region sequencing. ITS amplicons were purified using EuroClone^®^ spinNAker purification kit (Milan, Italy) and sent to an external provider (Eurofins Genomics, Vimodrone, Italy) for sequencing. The sequence analysis of the amplicons was performed by BLASTN comparisons in the National Center for Biotechnology Information (NCBI) database to confirm the identities of the selected strains.

ITS sequences were deposited at the NCBI database. GenBank accession numbers are reported in the Supplementary Materials (Table S1).

#### 2.2.4. Bacterial Identification

Bacterial isolates were initially screened using colony PCR to rapidly assess different profiles. Cultures streaked onto solid media (e.g., YEPD) were processed by preparing a DNA template, suspending it in 100 μL of TE buffer 1X (TRIS HCl 10 mM EDTA 1 mM, pH 8) heating the suspension to 98 °C for 10 min [25], and using the supernatant as DNA for amplification.

For samples where DNA extraction was not optimal, an alternative method was used. A single bacterial colony was suspended in 400 μL of TE 1X (TRIS-HCl 10 mM, pH 8; 1 mM EDTA), followed by the addition of 4 μL of lysozyme [50 mg/mL] and incubation at 37 °C for 30 min. Then, 12.5 μL of 20% (*w*/*v*) SDS and 10 μL of proteinase K [20 mg/mL] were added, followed by another 30-min incubation at 37 °C. The mixture was then treated with 400 µL each of phenol and chloroform and centrifuged at 13,680× *g* 10 min (Hettich, MIKRO 200, Tuttlingen, Germany) to separate nucleic acids. The aqueous phase was mixed with 40 μL of sodium acetate (3 M, pH 5.2) and 800 μL of 95% ethanol were added. The mixture was centrifuged at 18,620× *g* 30 min. The resulting DNA pellet was washed with 300 µL of 70% ethanol, centrifuged at 18,620× *g* 15 min, dried at 37 °C for 1 h, and resuspended in 50 µL of molecular-grade water before storage at −20 °C.

For ITS region amplification, the primers G1 (5′-GAAGTCGTAACAAGG-3′) and L1 (5′-CAAGGCATCCACCGT-3′) were used to target the bacterial 16S-23S rRNA gene spacer region. The PCR reaction, in a total volume of 25 µL, included 80–100 ng of bacterial genomic DNA, 2.5 µL of 1X PCR reaction buffer (GenScript, Piscataway, NJ, USA) containing 50 mM KCl, 10 mM Tris HCl, 1.5 mM MgCl_2_, and 0.1% Triton X-100. The reaction also contained 2.5 mM MgCl_2_, 0.2 mM dNTP mixture, 0.5 µM of each primer, and 0.5 U of Taq polymerase (GenScript, Piscataway, NJ, USA). The thermal cycling conditions were as follows: initial denaturation at 94 °C for 5 min, followed by 25 cycles of 94 °C for 1 min, 55 °C for 7 min, and 72 °C for 2 min. A final extension step at 72 °C for 7 min completed the PCR program. Positive and negative controls were included to ensure result accuracy. Following profile evaluation via agarose gel electrophoresis, the 16S rRNA gene was amplified using universal bacterial primers BSF8 (5′-AGAGTTTGATCCTGGCTCAG-3′) and BSR1541 (5′-AAGGAGGTGATCCAGCCGCA-3′). The PCR mixture was prepared as described above. The PCR conditions were: initial denaturation at 94 °C for 5 min, followed by 35 cycles of 94 °C for 1 min, 56 °C for 1 min, and 72 °C for 1 min, with a final extension at 72 °C for 5 min. Amplicons were stored at 4 °C upon completion. The quality of the amplicons was verified, followed by purification and sequencing, using methods consistent with those employed for fungal isolates.

16S rRNA genes sequences were deposited in the NCBI database. GenBank accession numbers are reported in Supplementary Materials (Table S2).

#### 2.2.5. Quantification of Isolation Frequency

The Isolation Frequency (*%IF*) of cultivable endophytes was calculated as follows:

%IF=(N/St)×100

where *N* represents the “presence” (1) or “absence” (0) of an isolate from the sample considered, and *St* indicates the total number of samples.

### 2.3. Microbiome Signature

#### 2.3.1. DNA Extraction

For culture-independent microbial community analysis, total DNA was extracted using a modified version of the CTAB method described by Murray and Thompson (1980) [26]. The study included two populations: *Vitis sylvestris* (wild) and *Vitis vinifera*, cultivated under four different management systems (organic, biological, biodynamic, and abandoned). The experiment was conducted in triplicate, processing three grapevine leaves from three different plants for each vineyard/population. Prior to DNA extraction, leaves were surface-sterilized as described above frozen in liquid nitrogen and pulverized using a sterile mortar. Briefly, 100–150 mg of leaves were ground with liquid nitrogen and suspended in 1 mL of preheated to 60 °C CTAB Buffer (3% (*w*/*v*) containing 0.2% β-mercaptoethanol. Samples were incubated at 60 °C for 1 h; then, 5 µL of RNAse (10 mg/mL) was added to the supernatant, and DNA extraction was performed twice using an equal volume of chloroform/isoamyl alcohol (24:1), followed by centrifugation at 18,620× *g* for 5 min. The DNA solution was then precipitated with an equal volume of isopropanol and incubated at −80 °C for 30 min. After centrifugation at 18,620× *g* 10 min, the supernatant was discarded and the pellet was rinsed with 70% ethanol, treated with 40 µL of 3 M sodium acetate, and dried at 50 °C for 15 min. Finally, the DNA was resuspended in 50 µL of TE 1X and incubated at 37 °C for 30 min to ensure complete solubilization.

#### 2.3.2. Library Preparation and MinION Sequencing

For library preparation, template DNA was quantified using the Qubit 4 Fluorometer (ThermoFisher Scientific, USA) to ensure a final concentration below 1000 ng. PCR amplifications were carried out in 25 µL reactions using Q5 Hot Start High-Fidelity DNA Polymerase (BioLabs, New England, Ipswich, MA, USA). Each reaction contained 5 µL Q5 Reaction Buffer [5X], 0.5 µL dNTPs [10 mM], 1.25 µL of both Forward and Reverse Primers [10 µM], 5 µL Q5 High GC Enhancer [5X] and 0.25 µL Q5 Hot Start High-Fidelity DNA Polymerase [0.02 U/µL]. Positive and negative controls were included in all reactions to ensure experimental validity. The 16S rRNA gene and ITS1-NL4 region for bacteria and fungi, respectively, were amplified. Primers and PCR conditions are summarized in Table 2.

PCR products were analyzed on 0.8% agarose gel (0.8% agarose, 0.4 mg/mL ethidium bromide, 0.5% TBE buffer Tris-borate-EDTA) under electrophoresis conditions of 100 V and 400 mA for 45 min. DNA bands were visualized using a transilluminator (Bio-Rad, Hercules, CA, USA). DNA purification was performed using AMPure XP beads (Beckman Coulter, Milano, Italy) according to the manufacturer’s protocol, with slight modifications. Briefly, 40 µL of AMPure XP beads were added to each adaptor-ligated DNA sample and incubated at room temperature for 5 min. Beads were then separated using a MagRack 6 magnetic stand (GE Healthcare, Uppsala, Sweden) for 3–5 min, and the supernatant was discarded. The bead pellet was washed twice with 200 μL of 70% ethanol for 1 min each, followed by drying at 37 °C for 3–5 min until residual ethanol evaporated completely. Finally, 40 μL of nuclease-free water was added directly to the bead pellet, incubated for 3 min at room temperature, and transferred to a new tube, discarding the beads.

All subsequent quantifications were performed using a NanoDrop 1000 (Thermo Scientific, Wilmington, MA, USA). A total of 200 fmol of each sample was used for the barcoding step, according to the ligation sequencing kit 1D (SQK-LSK109) and the PCR barcoding expansion pack 1–96 (EXP-PBC096) protocols (Oxford Nanopore Technologies, Oxford, UK). After purification, barcoded libraries were pooled to achieve a final concentration of 1 μg of DNA in 47 μL of nuclease-free water. The library was then prepared for Nanopore sequencing using the NEBNext Ultra DNA library preparation kit. The final product was quantified to concentration of 50 fmol and loaded onto a R9.4.1 flow cell.

Reads were base-called on-instrument using the Guppy v.4.2.2 GPU base caller (Oxford Nanopore Technologies), with the option *-min_qscore* 20 to filter out reads with a quality score below 20. The sequence analysis pipeline was executed in a Conda environment on Ubuntu. Raw reads were filtered using *seqtk* to remove sequences shorter than 400 bp and longer than 1800 bp. Filtered reads were merged into a single file, which served as input for the alignment program minimap2 (version 2.24). Fungal identification was performed by aligning sequences against the UNITE General Release reference database from (version sh_general_release_04.04.2024.tgz) and the Silva LSU database (SILVA_138.1_LSURef_tax_silva.fasta). Metabarcoding analysis for Prokaryotic identification was conducted by mapping filtered raw reads against SILVA 16S database (SILVA_138.1_SSURef_tax_silva.fasta.gz). The mapping algorithm was tuned to support the alignment of long-noisy reads by using the option map-ont, which uses ordinary minimizers as seeds. Results were stored in SAM files and processed with SAMtools package to generate tab-delimited tables. The final output consisted of a list of reference strains with the corresponding number of mapped raw reads.

Genes sequences were deposited at the NCBI database. GenBank accession numbers (Table S3), along with the quantity and quality metrics of raw and filtered sequencing data (Table S4), are reported in the Supplementary Materials.

### 2.4. Statistical Analysis

#### 2.4.1. Semi-Quantitative

Detection frequency (*%DF*) was used to evaluate the occurrence of times a strain was recorded across all samples. It was calculated by converting abundance matrices into binary tables where 1 indicated “presence” and 0 indicated “absence”. The frequency was then obtained by counting the occurrence of 1 (*P*) divided by the total number of samples (*T*).

%DF=(P/T)×100

A one-way ANOVA test was applied to evaluate the effect of geographical origin, cultivation method, *Vitis* species, and tissue type on microbial diversity. Identification values obtained through the cultivable approach were recorded as presence/absence data, where a value of 1 was assigned to presence and 0 to absence. For factors showing a statistically significant difference (*p*-value < 0.05), a Tukey test (HSD) was performed, comparing means with a 95% confidence interval.

#### 2.4.2. Quantitative

Data were normalized using the Geometric Mean of Pairwise Ratios (GMPR) via the trans_norm function from the R package *microeco*. Microbial diversity was assessed using alpha and beta diversity metrics. Alpha diversity was measured using the Shannon diversity index, Simpson Evenness index, and Chao richness index. Beta diversity was evaluated with Bray–Curtis index and visualized through seven different ordination methods: Detrended Correspondence Analysis (DCA), Canonical Correspondence Analysis (CCA), Redundancy Analysis (RDA), Double Principal Coordinate Analysis (DPCoA), Non-metric Multidimensional Scaling (NMDS), Metric Multidimensional Scaling (MDS), and Principal Coordinate Analysis (PCoA). All diversity analyses were performed using the *Vegan* and *microeco* packages in the R environment. To visualize microbial composition, bacterial and fungal genera were represented through donut and stacked column charts, grouped by *Vitis* treatment. Radar charts were used to compare the abundance of these genera across geographical locations. A differential abundance analysis was conducted to identify taxa that significantly contributed to community differences between groups. This analysis was carried out using the “object$res_diff” function from the *microeco* package in R. Core microbiome analysis was performed using the “core” function from the *Microbiome* package, identifying core taxa at varying prevalence thresholds.

## 3. Results and Discussions

The obtained results demonstrated the successful development of culture techniques for isolating grapevine endophytes. The choice to consider different grapevine species, geographical origins, and vineyard management practices in the cultural approach, followed by the metabarcoding analysis conducted on leaf samples provided a detailed overview of the microbial biodiversity associated with the plants. This comparative analysis of identification techniques not only offered insights into the microbial communities present but also highlighted the strengths and limitations of each method.

### 3.1. Endophytic Population Isolated with Culture-Dependent Methods and Their Identification

The growing interest in the role of endophytes has necessitated the development of effective sterilization protocols for different parts of the grapevine [27]. Studies have shown that sodium hypochlorite [2–10%] and ethanol [70–90%] are useful sterilizing agents, and their efficiency can be further enhanced by pre-treating the samples with surfactants, such as Triton X-100, Tween 80, and Tween 20 [28]. Determining the optimal concentration of these compounds to avoid damage to plant material, while preserving the endophytic microbial population, is a critical step in laboratory procedures. To address this, challenge tests using *S. cerevisiae* as the target microorganism were conducted to assess the efficacy of the sterilization treatment. According to the Section 2, two different sterilization protocols (P1 and P2) were applied. As expected, monitoring of the pre-sterilization wash water revealed the presence of numerous bacterial and fungal colonies, confirming initial contamination on the plant material surface. Due to the heterogeneous growth of bacterial and fungal colonies, a qualitative assessment of microbial presence on grape surfaces was performed. The use of *S. cerevisiae* resulted in contamination levels of approximately 1.7 × 10^7^ cells/mL for shoots and 1.8 × 10^7^ cells/mL for berries, with a standard error of 10% among replicates. The most effective sterilization protocol was P1, which resulted in no colony proliferation after plate incubation (<1 cell/mL). Overall, it proved effective even at *S. cerevisiae* contamination levels up to 10^7^ cells/mL. The results indicated that leaves, shoots, and grape berries could all be treated using the same protocol without compromising their structure. Notably, the more sensitive berries showed no visible damage to their exocarp, whose alteration could have affected the endophyte population. Based on these findings, protocol P1 was selected for sterilizing all plant parts in this study.

The assessment of endophytic communities was initially conducted using a culture-dependent method, which involved isolating morphologically different colonies from grape berries, leaves, and shoots, followed by molecular identification. A total of 148 endophytes were isolated: 41 from shoots, 94 from leaves, and 13 from berries. Microscopic analysis classified them into 42 fungal and 106 bacterial strains.

**Figure 1:**
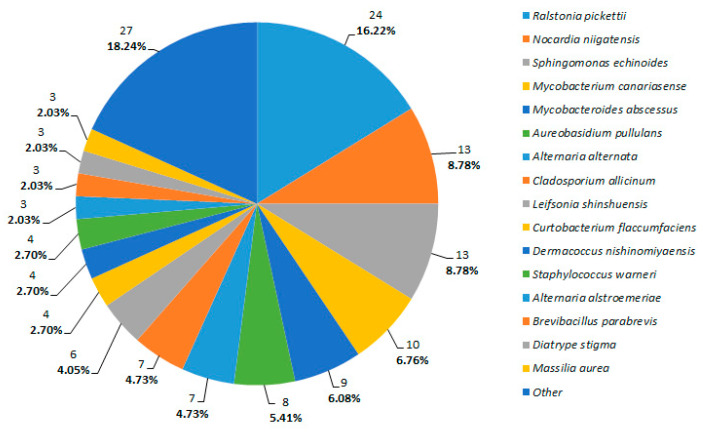
The percentage of most frequently cultivable isolated endophytes’ species and in brackets the number of the corresponding isolates The frequency of detection (DF) was used to evaluate the number of times a strain was recorded across all samples (presence/absence).

Sequencing revealed that among the fungal populations, the most frequently isolated species were *Aureobasidium pullulans* (5.41%), *Alternaria alternata* (4.73%), and *Cladosporium allicinum* (4.73%) (Figure 1). The presence of *Aureobasidium* ssp., *Alternaria* ssp., and *Cladosporium* ssp. as cultivable fungal endophytes has been confirmed in multiple studies, highlighting their prevalence in grapevines [29,30]. *Cladosporium* spp. in grapevine may play a role in bioactive metabolite production, as they are involved in the production of the anticancer enzyme L-asparaginase in *Asteraceae* family [31]. *Alternaria* spp. typically dominate endophytic communities in phyllospheres, and are characterized by the production of highly melanized hyphae able to grow under intense UV radiation [32]. *A. alternata* is commonly considered an ubiquitous filamentous pathogen causing black rot in a wide variety of fruits and vegetables. However, it has also been studied as a potential biocontrol agent, as it can inhibit the sporulation of *P. viticola* through the production of a low molecular weight metabolite [33]. These findings suggest that endophytic and pathogenic fungi can coexist in plant tissues, leading to the hypothesis that the absence of symptoms related to downy mildew infection in the sampled plants, such as leaf lesions appearing as yellow spots on the upper leaf epidermis and white fungal sporulation on the lower epidermidis, might be correlated with the presence of this beneficial endophyte in the population.

*Aureobasidium pullulans* is frequently found in various phyllospheric environments, including grapevines, and exhibits significant morphological and genetic diversity [34]. It is one of the predominant yeast species isolated from diseased and healthy vines, particularly from grape berries at all stages of maturity. It has demonstrated biocontrol capabilities against pathogens such as *B. cinerea* [35] and *D. seriata* [36]. The role of endophytic fungi in relation to phytoplasma infection and spontaneous recovery from disease symptoms remains to be explored, but their presence can impact the microbial community dynamics by inhibiting the establishment of pathogens [37].

Regarding bacteria, the most frequently isolated species was *Ralstonia pikettii* (16.22%), followed by *Nocardia niigatensis* and *Sphingomonas echinoides* (8.78%), and *Mycobacterium canariasense* (6.76%) (Figure 1). *Ralstonia pickettii* has been identified as a dominant endophytic bacterium in grapevines and it is recognized as a plant growth-promoter (PGPB) due to its phosphate-solubilizing activity [38]. This genus can survive in wine fermentation environments, though its impact on grape quality and fermentation processes remains unclear [39]. Other bacteria isolated in this study, albeit with lower isolation frequency, have been described as grapevine endophytes. *Curtobacterium* sp., *for instance*, can induce systemic resistance (ISR) in other plant hosts [40], while *Pantoea agglomerans* [41], inhibits *N. parvum*, a causative agent of grapevine trunk diseases (GTDs), by secreting antifungal volatile compounds [42].

#### Endophyte Prevalence in Different Grapevine Organs Under Various Vineyard Management Practices

**Figure 2:**
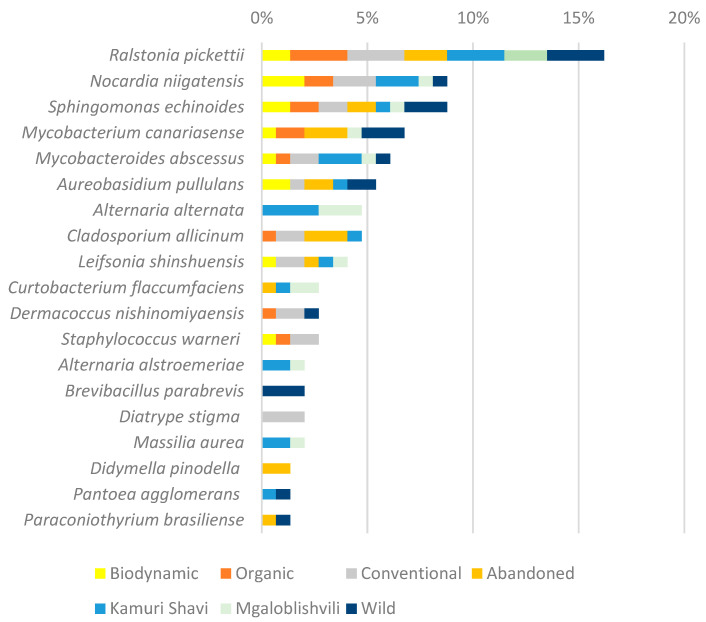
Percentage of principal cultivable endophyte species isolated from different vineyards/populations. Detection frequency (DF) was used to evaluate the number of times a strain was recorded across all samples (presence/absence).

In terms of the geographical origin of the samples and/or the management system employed, *A. alternata* was predominantly found in shoots collected from the Georgian cultivars *Mgaloblishvili* and *Kamuri shavi* in Riccagioia. Meanwhile, *C. allicinum* was isolated from all analysed plant materials obtained from vines in Franciacorta, which were grown under conventional, organic, and abandoned cultivation methods, as well as from resistant cultivars in Riccagioia. *A. pullulans* was detected in shoots and grape berries sampled from biodynamic, conventional, and abandoned vineyards in Franciacorta, from *Kamuri Shavi* in Riccagioia and from the *V. sylvestris* population in Monte Fenera (Figure 2).

Wijekoon and collaborators (2021) [19] observed that *Alternaria* sp. and *Cladosporium* sp. were the dominant culturable strains isolated from table grapes, affirming their consistent presence as endophytes in grapevines regardless of the vineyard management system. These genera are prevalent endophytes in grape leaves and fruits across different regions, where they act as considerable pre- and post-harvest pathogens in table grapes and various fruit tissues, such as strawberries and mangroves [43].

**Figure 3:**
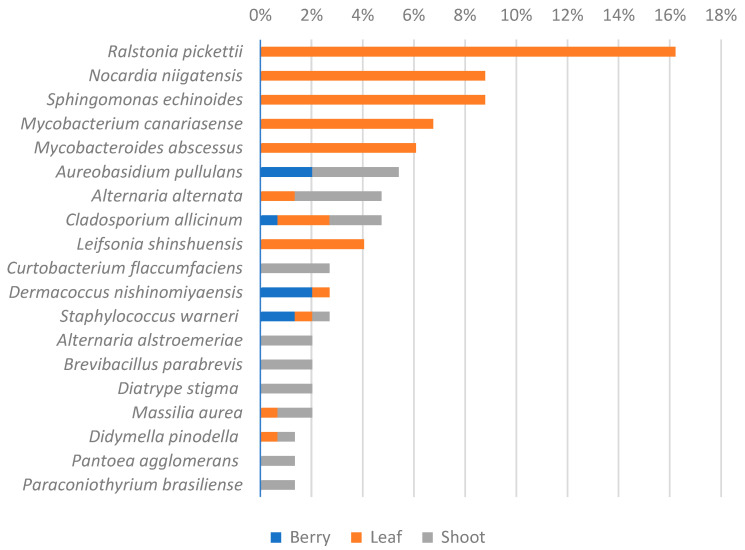
Percentage of principal cultivable endophyte species isolated from different plant organs. Detection frequency (DF) was used to evaluate the number of times a strain was recorded across all samples (presence/absence).

*Ralstonia pickettii* was isolated across all sampled locations (Franciacorta, Riccagioia, Montefenera), regardless of the cultivation methods or the plant species (wild or domesticated). The four most frequently isolated bacteria were predominantly found in leaf samples (Figure 2). In other plant materials, *Curtobacterium flaccumfaciens* and *Brevibacillus parabrevis* were frequently isolated from abandoned fields, resistant cultivars, and *V. sylvestris* shoots, while *Dermacoccus nishinomiyaensis* and *Staphylococcus warneri* were common in grape berries from organic, conventional, and biodynamic vineyards (Figure 3).

Campisano et al. (2014) demonstrated that high levels of the dominant *Ralstonia* genus were detected in all the grape samples in their study [10]. This finding confirms that this genus is typically present in grapevines, and its occurrence does not depend on the cultivar or vineyard management approach, suggesting that it could be a permanent component of the plant’s microbial community.

Statistical analysis was performed to evaluate whether *Vitis* species, geographical origin, tissue type, or vineyard management influenced microbial frequencies. The ANOVA test showed a statistically significant difference between tissue types with a *p*-value < 0.05 with leaf samples differing significantly from grape berry and shoots; the Tukey HSD test confirmed this result. These findings align with those reported by Deyett et al. [13], who studied bacterial and fungal populations across different grapevine tissues and revealed distinct microbial diversity and composition across tissues such as root, rhizosphere, cane, cordon, and sap, while also identifying a core group of taxa present throughout the vine, both above and below ground. Additionally, Campisano et al. [43] firstly highlighted a significant difference in bacterial endophyte populations between wild and domesticated grapevines, identifying 118 strains belonging to 25 genera in the former compared to only 37 strains from 6 genera in the latter.

In conclusion, it is possible to hypothesize that microbial composition in grapevines varies qualitatively and quantitatively across different tissues in terms of relative abundance. However, despite these variations, a core microbiota appears to be shared among all tissues, but in different proportions. Microbes may use the host vascular system to move within the endosphere, positioning themselves in specific compartments of the plant and forming microbial niches in certain tissues where conditions are most favourable for their growth and survival.

### 3.2. Metabarcoding Analysis of Endophytic Community

Microbial biodiversity analysis was conducted using a non-cultivable approach, providing a more comprehensive picture of the endophytic community present in grapevines, specifically within the leaf tissue. The results regarding the quantity and quality of the new generation of raw and filtered sequencing data are shown in the Supplementary Materials (Table S4).

For the metabarcoding analysis, amplification of the ITS and 26S regions was performed for fungi, and the 16S rRNA gene for bacteria. The limited variability of ITS regions is well known, and it may impair the discrimination of closely related taxa, particularly when classifying fungi. Barcoding must be associated with specific fungal taxa, therefore, defining a single barcode for all fungal species is impossible [44]. Additionally, identification was performed at the genus level, as the commonly used 97% similarity threshold for sequence clustering is generally insufficient for differentiating closely related taxa. Increasing the similarity threshold to 99% may introduce additional challenges without significantly improving taxonomic resolution [45]. It is recommended to target fungi using the 26S rRNA region, as it provides a higher alpha diversity index and greater taxonomic robustness of fungal rRNA compared to ITS2. ITS2 has been shown to underestimate species with longer fragments while overestimating those with shorter fragments. Therefore, combining both target regions is suggested, using ITS for species identification and 26S for phylogenetic analysis, depending on the research objectives [46]. Similar challenges apply to 16S rRNA gene sequencing for bacterial identification, where conserved regions often limit species-level identification [47]. Culturing endophytes is a simple and cost-effective method essential for understanding their ecological roles and potential applications, despite its limitations in capturing the full diversity of microbial communities. Traditionally, culture-dependent techniques were principal methods for isolating endophytes from plant tissues, consisting of surface sterilization followed by cultivation on appropriate media [48]. However, total microbial diversity cannot be fully assessed using culture-dependent techniques alone due to the specific growth requirements of certain endophytes, or the inability of some to grow on solid media. Therefore, these techniques are complemented by metabarcoding analyses, which provide a more comprehensive view of microbial diversity by including non-cultivable species.

#### 3.2.1. Bacterial Diversity Across Vineyard Practices in Grapevine Leaves

**Figure 4:**
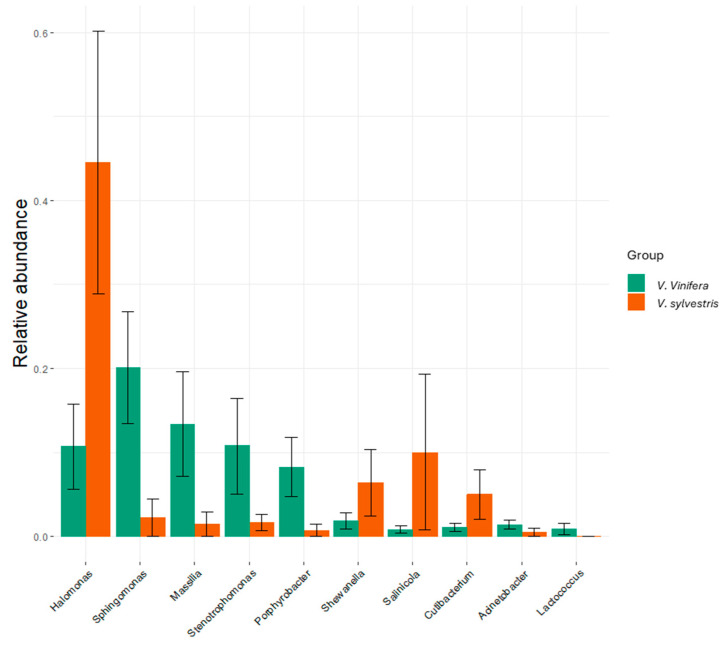
Comparison of the relative abundance of bacterial endophyte genera between wild (*V. sylvestris*) and domesticated (*V. vinifera*) grapevine leaf samples, based on 16S rRNA gene identification.

Comparing the relative abundance of bacterial species (Figure 4) detected in *V. sylvestris* and *V. vinifera* samples, *Halomonas* was the prevalent genus in wild *V. sylvestris*, accounting for 44.6% of the average abundance, followed by *Salinicola* (10%). In contrast, *Sphingomonas* was the most prevalent genus in *V. vinifera* (20%), followed by *Massilia* (14%). Halophilic bacteria, such as *Halomonas*, have previously been associated with salt-tolerant plants, promoting plant growth [49]. Marzano et al. (2016) also identified this genus in grape must throughout fermentation [50]. Another possible explanation for the presence of *Halomonas* on grapevines is the whitefly, *Bemisia tabaci*, an agricultural pest known to interact with grapevines [51]. Wang et al. (2021) reported *Halomonas* species in the whitefly’s midgut, suggesting that the insect could act as a vector, transferring *Halomonas* from its microbiome to the grapevine [49]. This vector-mediated mechanism may explain the presence of *Halomonas* in *V. sylvestris*, especially in regions with high *B. tabaci* populations. West et al. (2010) highlighted that bacterial endophyte populations are both highly homogenous and unique to each vine and location, illustrating the complex relationships between plants and their environments [14]. On the other hand, in domesticated grapevine groups, *Sphingomonas* had the highest average abundance, around 20%. Leveau and Tech (2010) found *Sphingomonas* to be the most abundantly represented genus on grapevine leaf tissue (9.5%) through 16S rRNA gene analysis [52]. Other high-throughput sequencing studies, such as those by Yang and co-authors (2024) and Ding et al. (2021), also identified *Sphingomonas* as a major bacterial genus in grape tissues [38,53].

**Figure 5:**
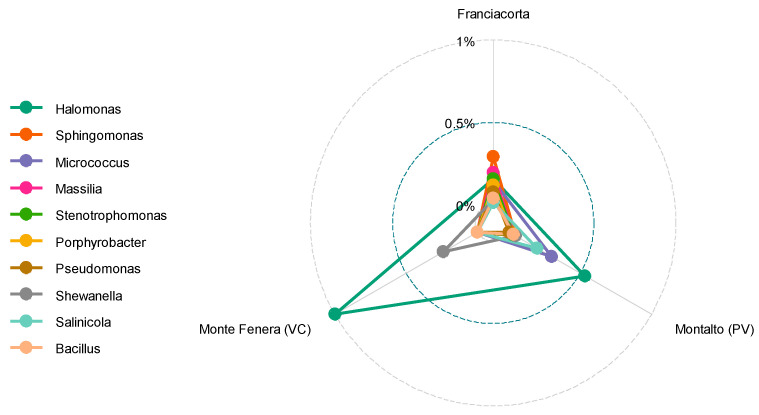
Comparison of the relative abundance of identified bacterial genera across different geographical locations in grapevine leaf samples.

All *V. vinifera* samples were collected from the winemaking area of Franciacorta (Lombardy, Italy), while *V. sylvestris* samples were gathered from both Monte Fenera (VC, Piedmont) and Montalto (PV, Lombardy). Examining the relative abundances of species based on sampling locations, *Halomonas* exhibited higher relative abundance in Monte Fenera compared to Franciacorta and Montalto (Figure 5). This suggests that environmental conditions at Monte Fenera, including soil salinity, pH, moisture, and nutrient availability, may promote its growth. The area is characterized by sedimentary rocks, including pre-Cambrian gneiss schists, quartziferous porphyries, and substantial calcareous-dolomitic rock layers. Additionally, historical marine influence during the Pliocene has left marly deposits rich in marine salts and minerals (Parco Naturale Del Monte Fenera, accessed 2024). Bokulich and collaborators (2014) linked microbial diversity in grapevines to different geographical locations and crop conditions, particularly noting the intense environmental interactions of wild grape plants [39]. Guzzon et al. (2023) suggested that while some taxa are consistently present across locations, variations in relative abundance are observable in different areas [54].

**Figure 6:**
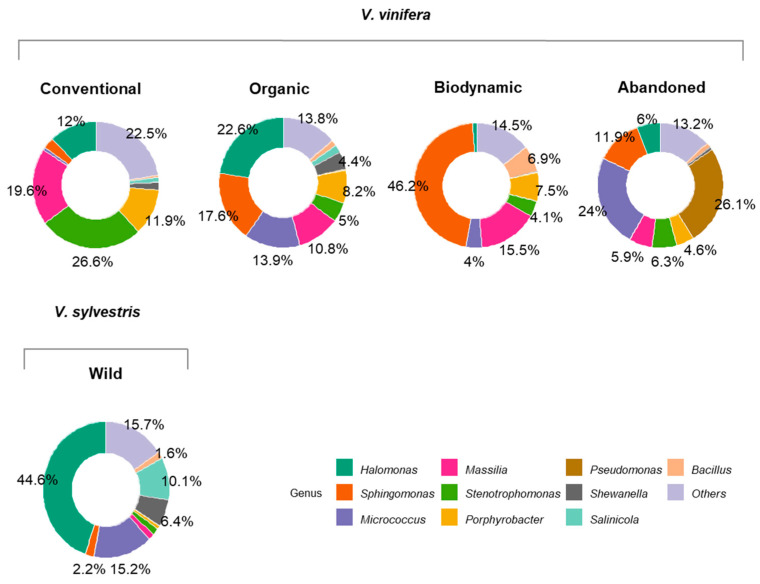
Comparison between wild (*V. sylvestris*) and domesticated (*V. vinifera*) 16S rRNA gene identification from grapevine leaf samples, separated by farming conditions (conventional, biodynamic, organic, and abandoned). Percentages indicate the relevant average abundance of the genus within each group.

Different vineyard management practices have led to variations in bacterial communities (Figure 6). In grape leaf samples from conventional farming, *Stenotrophomonas* was the most abundant genus, with an average abundance of 26.6%, followed by *Massilia*, at 19.6%. In biodynamic farming samples, *Sphingomonas* was the most prevalent genus, accounting for approximately 46.2% of the bacterial community, with *Massilia* also significant at 15.5%. Organic farming samples were dominated by *Halomonas* (22.6%), *Sphingomonas* (17.6%), and *Micrococcus* (13.9%). In contrast, samples from abandoned fields were primarily composed of *Pseudomonas* (26.1%) and *Micrococcus* (24%). As previously indicated, *Halomonas* was the most abundant genus in *V. sylvestris* leaf samples, with an average abundance of 44.6%, followed by *Micrococcus* at 15.2%.

**Figure 7:**
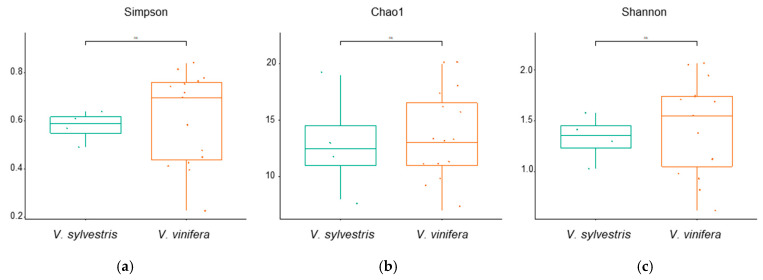
Analysis of the sample alpha-diversity: (**a**) Simpson index, *p*-value: 0.02898; (ANOVA) F-value: 3.4793; (**b**) Chao1 index, *p*-value: 0.012439; (ANOVA) F-value: 4.339; (**c**) Shannon index, *p*-value: 0.004172; (ANOVA) F-value: 5.5182. The colored solid line inside the box is the median value; the colored dots are individual samples. Non significant differences were indicated with the term “ns”.

Alpha and beta diversity analyses were conducted to assess microbial ecosystem structure and complexity. Alpha diversity assesses the diversity within a single community, capturing aspects such as species richness, evenness (relative abundance), and taxonomic distribution [55]. In this study, the main alpha diversity indices were used to presents results for the two different *Vitis* species considered. *V. vinifera* exhibited higher diversity compared to *V. sylvestris*, but these differences were not statistically significant (Figure 7).

**Figure 8:**
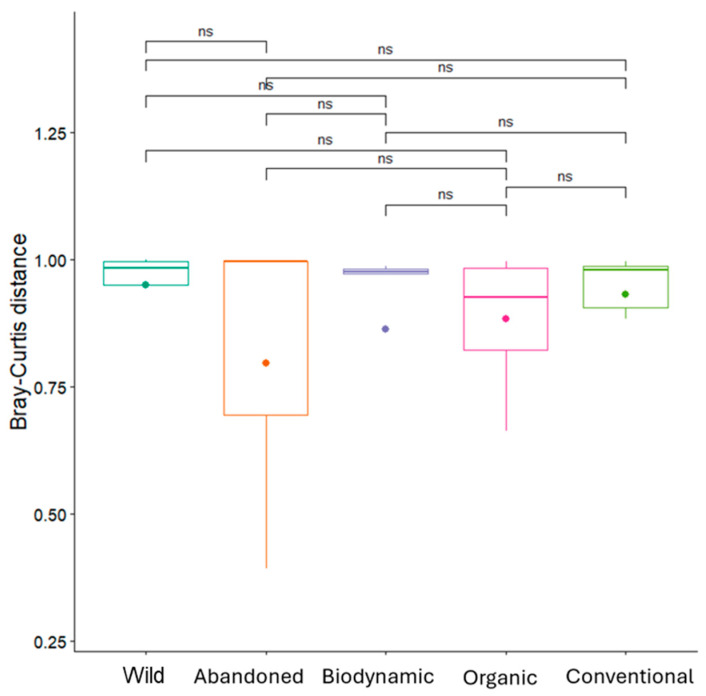
Beta diversity of bacterial samples. Y–axis: Bray–Curtis distance, a measure of dissimilarity between microbial communities; X–axis: Different treatments/populations (wild, abandoned, biodynamic, organic, and conventional). Non significant differences were indicated with the term “ns”.

**Figure 9:**
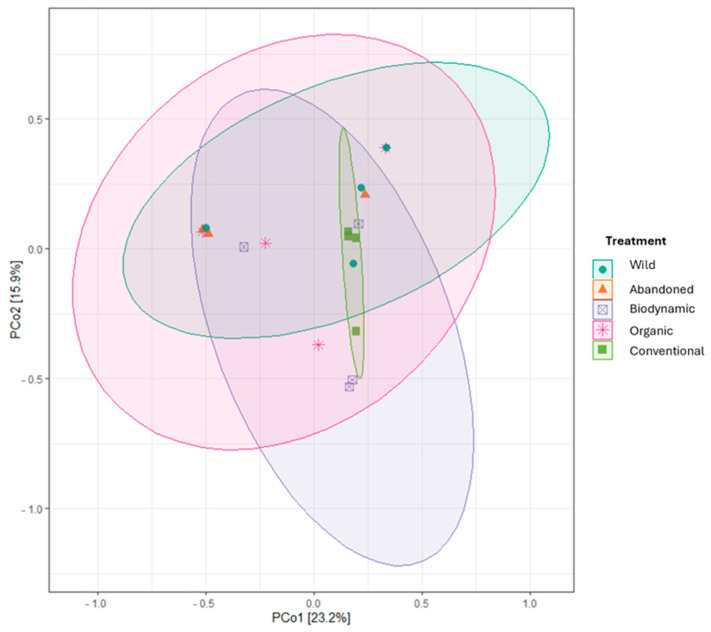
Principal Coordinates Analysis (PCoA Plot). Axes: PCo1 and PCo2 are related to 23.2% and 19.3% of the variance, respectively. Points: represent samples, color-coded and shaped according to the population/condition treatments (wild, abandoned, biodynamic, organic, and conventional). Ellipses: represent 95% confidence intervals for each treatment.

Beta diversity assesses compositional differences between communities [56]. The non-significant Bray–Curtis distances (Figure 8) and the overlapping ellipses in the PCoA plot (Figure 9) indicate that microbial community composition does not significantly differ across the various agricultural practices. Overall, farming conditions did not lead to substantial changes in the primary composition of bacterial communities in grapevine leaves, a hypothesis supported by a MANOVA statistical test (Table S5). These results align with those reported by Aleynova et al. (2022), who conducted a study on wild grape *V. amurensis* and domesticated *V. vinifera* cultivars, revealing that both exhibit the same basic composition of endophytic bacteria detected by NGS approach [57].

##### Comparison Between Metabarcoding and Culture-Dependent Approach for Bacterial Population

**Figure 10:**
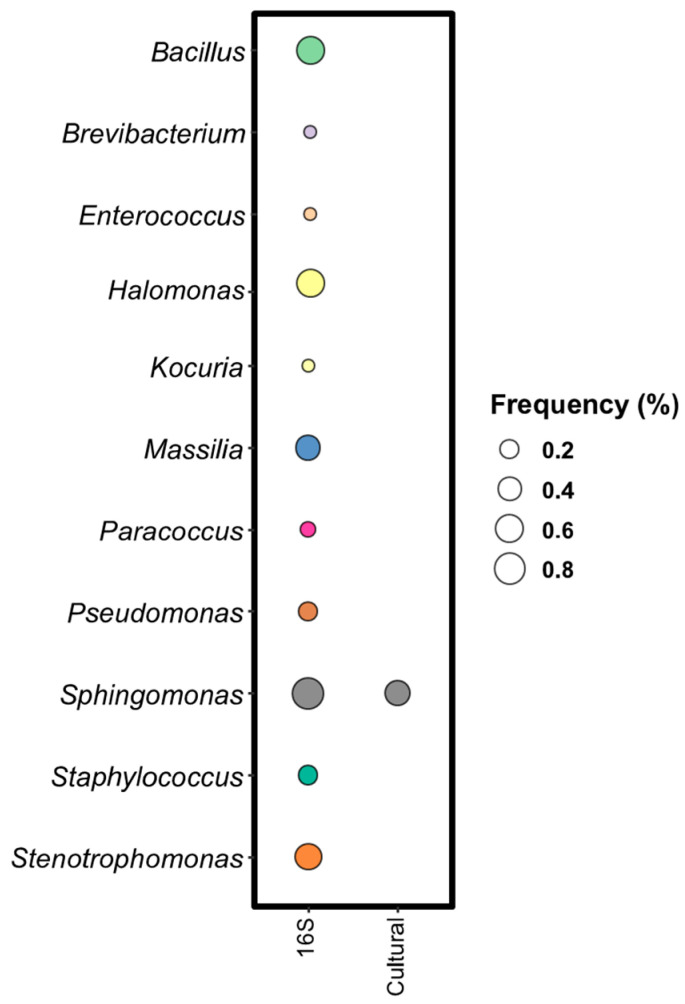
Common endophytes found with 16S rRNA gene and culturable isolated. The columns indicated with genetic marker (16S rRNA gene) report the Detection frequency (DF) at the genus level, while the column designate with “Cultural” shows the identification frequency (IF). Bubbles diameter is proportional to the calculated frequencies: the bigger the diameter, the higher the frequency.

Bacterial identification revealed a total of nine different species in vine leaves using the culture-dependent method, with *R. pickettii* (73.68%), *S. echinoides* (47.37%), *N. niigatensis*, and *M. abscessus* (42.11%) being the most common. Metabarcoding analysis was conducted at the genus level using 16S rRNA gene sequencing, identifying 78 different species, with *Sphingomonas* being the most prevalent, present in 78.95% of the samples. Notably, *Sphingomonas* was the only endophytic bacterium identified by both cultivation and metagenomic approaches (Figure 10). As shown in different studies [58,59], including this work, a comparison between the consortium of cultivable endophyte genera and the metagenomic profile of grapevine leaves reveals that the presence of a genus can differ between the two approaches. Several factors may contribute to this discrepancy, such as presence of microbial communities that cannot be cultivated under laboratory conditions or methodological artifacts that create a knowledge gap between what is observed through bioinformatics methods and the species that can actually be cultivated [60]. However, it is important to underline that the microbial ecosystem is a highly complex network that involve dynamic spatial-temporal interactions between microorganisms and the environment where they live. Next-generation sequencing (NGS) has significantly advanced our understanding of plant-associated microbial communities, particularly endophytes, but challenges remain. Every step in the NGS process, from DNA extraction to data analysis, can impact the final results [61]. The choice of DNA extraction methods and sterilization protocols is crucial for obtaining high-quality sequences that accurately represent the microbial community. Additionally, the reference databases currently used for computational analysis may be incomplete or contain incorrect sequence annotations. To address this issue, regular updates and quality controlof databases is necessary in order to enhance the accuracy of data analysis [44,60]. 

#### 3.2.2. Fungal Diversity Across Vineyard Practices in Grapevine Leaves

**Figure 11:**
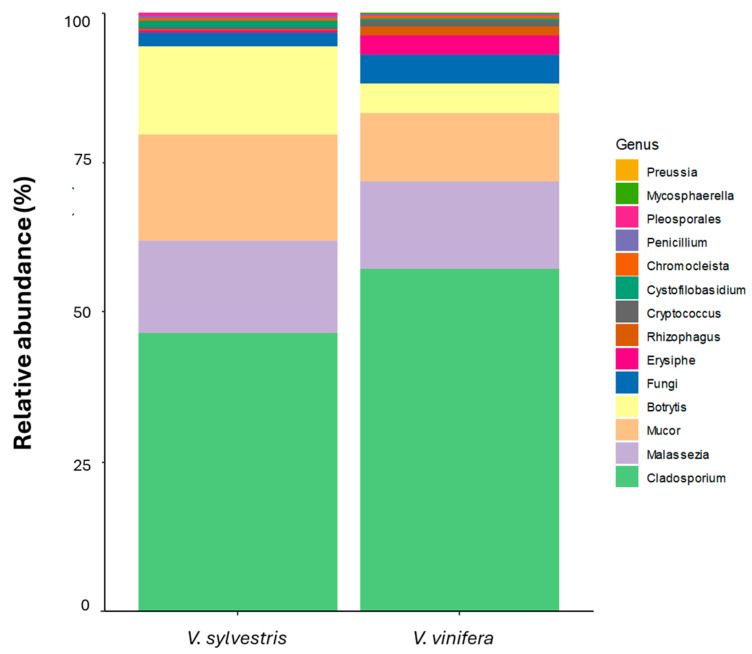
Relative abundance comparison of fungal genera between wild (*V. sylvestris*) and domesticated (*V. vinifera*) grapevine leaf samples.

Considering the fungal genera detected by the two databases and comparing the relative abundance of fungal species (Figure 11) between *V. sylvestris* and *V. vinifera* samples, *Cladosporium* was the most prevalent genus in both groups. In *V. vinifera* samples, *Malassezia* was the second most prevalent genus, followed by *Mucor*. Conversely, in *V. sylvestris* samples, *Mucor* was the second most prevalent genus, followed by *Malassezia* and *Botrytis*. Several studies have shown that these dominant genera are typically associated with wine, grapes, and leaves [17,27,62], confirming that they are true grapevine endophytes and may be part of the grapevine’s core microbiota. The core microbiota is defined as a set of species consistently found within specific compartments across various plants, regardless of genotype, age, geographic location, or environmental conditions such as soil type and climate [63].

**Figure 12:**
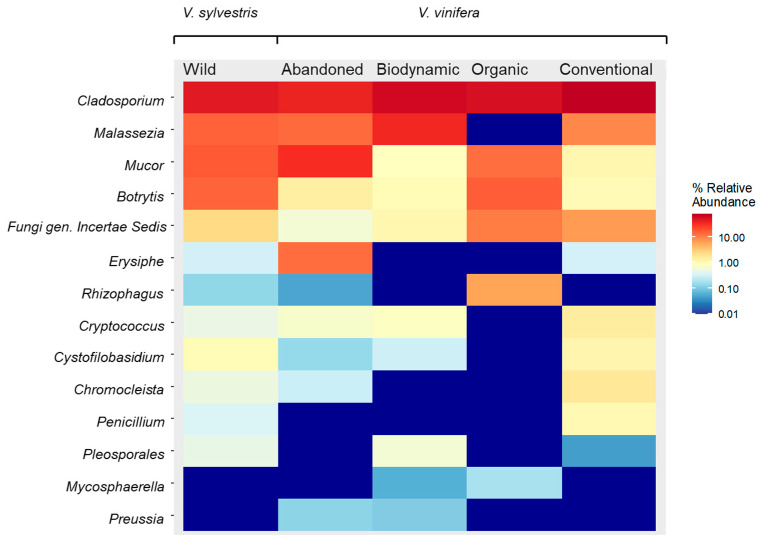
Heatmap showed the comparison between wild (*V. sylvestris*) and domesticated (*V. vinifera)* fungal genera identification from grapevine leaf samples, separated by populations/farming conditions (wild, conventional, biodynamic, biologic, and abandoned). Color of each cell indicates the relative abundance threshold. Red/orange: high abundance. Blue: very low or no abundance. Detrended correspondence analysis (DCA) (Figure 13) revealed that certain fungal genera (such as *Erysiphe*, *Malassezia*) appear to be more closely associated with *V. vinifera* while others, such as *Cladosporium*, exhibited a broader distribution between the two *Vitis* species.

In the heatmap (Figure 12), the fungal populations in different *V. vinifera* vineyards were compared with those in *V. sylvestris*. Grape leaf samples from all farming conditions analyzed showed *Cladosporium* as the most abundant genus, with an average abundance greater than 10%, followed by *Malassezia* and *Mucor*. In organic farming, *Mucor* was the second most abundant genus, while *Malassezia* was not present due to its low abundance.

**Figure 14:**
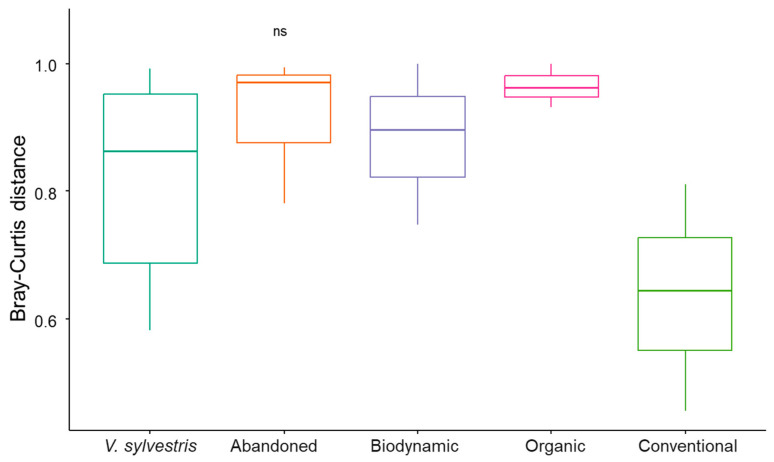
Beta diversity of fungal samples. Y–axis: Bray–Curtis distance, a measure of dissimilarity between microbial communities; X–axis: different treatments (abandoned, biodynamic, organic, and conventional). Non significant differences were indicated with the term “ns”.

The values derived from the Beta diversity analysis (Figure 14) were plotted using various ordination methods to corroborate these findings, demonstrating no significant separation among the different conditions (Figure S1). This suggests that cultivation techniques do not influence the fungal diversity associated with the two grapevine species.

In contrast to findings from other studies [10,12], this work did not reveal statistically significant differences in microbial community composition across different management systems. However, it is important to note that previously reported differences were primarily related to variations in abundance rather than overall composition. These findings suggest that the core microbial community remains stable, with management practices influencing the relative abundance of specific taxa rather than fundamentally altering the core microbiota.

These results challenge the common assumption that agricultural treatments typically reduce microbial diversity. Instead, they indicate that the endophytic environment of grapevines is relatively resilient to external factors, suggesting the existence of a stable core microbiota within the endophytic grapevine population.

##### Comparison Between Metabarcoding and Culture-Dependent Approach for Fungal Population

**Figure 15:**
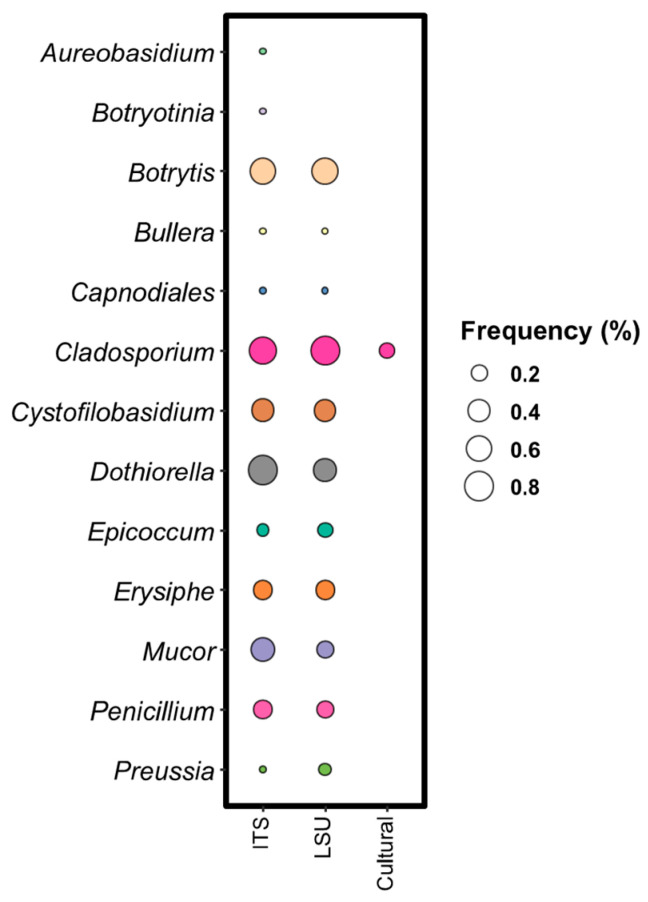
Common endophytes found with both ITS and LSU databases compared to culturable isolates. The columns indicated with genetic marker (ITS and LSU) report the detection frequency (DF) while the column designate with “Cultural” shows the identification frequency (IF) at genus level. Bubbles diameter is proportional to the calculated frequencies: the bigger the diameter, the higher the frequency.

Four fungal species were identified in grapevine leaves using the culture-dependent method, with *C. allicinum* (10.53%) being the most frequently detected, followed by *Didymella pinodella*, *Elsinoe salicina*, and *Talaromyces amestolkiae* (5.26%). Metabarcoding analysis at the genus level identified 72 and 105 different species using ITS and LSU databases, respectively. For ITS, *Cladosporium* and *Dothiorella* were the most frequent genera, each found in 83.33% of samples, while LSU analysis revealed a prevalence of *Cladosporium* (88.89%) and *Botrytis* (66.67%) (Figure 15). Common endophytes found in both ITS and LSU profiles included *Botrytis*, *Cladosporium*, and *Dothiorella*. However, species such as *Aureobasidium* and *Botryotinia* were detected only in the ITS primer database and in LSU. A combination of the 26S rRNA (D4 domain) and ITS1 regions is recommended for comprehensive species identification and phylogenetic analysis [64], as it allows for distinctionsbetween the discriminative capabilities of the two databases. Notably, *Cladosporium* was the only common genera detected in both culture-dependent and culture-independent approaches. The differences observed between the genera identified in fungal identification methods mirror those seen in the bacterial analysis, highlighting the challenges of integrating both approaches. These differences suggest that factors such as the inability of certain fungi to grow under laboratory conditions or limitations and inconsistencies in reference databases can influence fungal detection and identification. While both methods provide valuable insights into fungal communities, their observed differences underscore the complexity of accurately capturing the full spectrum of endophytic diversity. Therefore, combining both techniques is essential for a comprehensive evaluation of microbial biodiversity, as each approach offers unique advantages that, when integrated, provide a more complete picture of microbial diversity.

#### 3.2.3. Core Microbiome

Metabarcoding analysis assessed bacterial and fungal diversity across various sample groups, including wild (*V. sylvestris*) and domesticated (*V. vinifera*) species, different farming practices (conventional, biodynamic, organic, abandoned, and wild), and geographical locations (Monte Fenera, Montalto, and Franciacorta).

**Figure 16:**
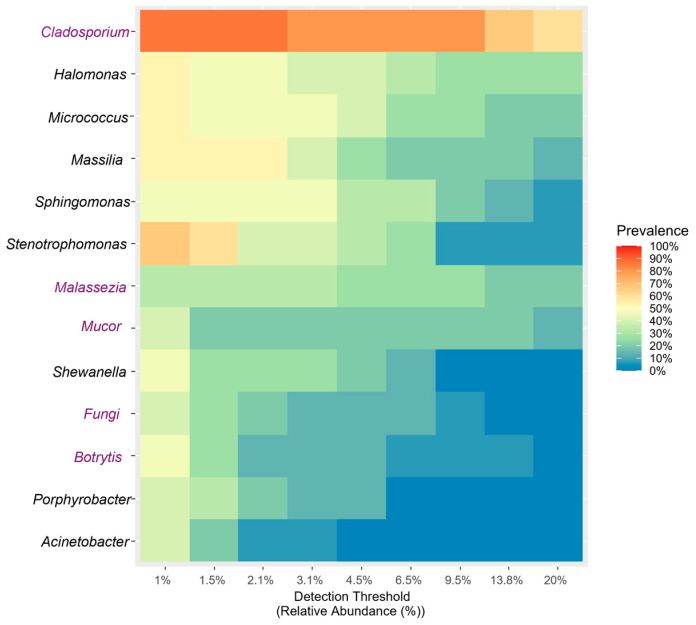
Heatmap graph. Row: microbial genus; column: detection threshold. Color of each cell indicates the prevalence of a given microbial genus at that particular abundance threshold. Red/orange: high prevalence. Blue: very low or no prevalence.

Since no significant differences were found in microbial composition among the factors considered, the presence of microorganisms consistently detected across the samples was evaluated. The results indicated that the core microbiota, defined as the set of microbial taxa characteristic of a host or environment of interest [65], is relatively stable. This stability suggests that endophytes are minimally affected by external variations [66]. The core microbiome plot (Figure 16) illustrates the proportion of samples (prevalence) in which a particular taxon is detected, given a specific detection threshold (the minimum relative abundance value at which taxa are considered “present” in compositional data). This provides an overview of how the prevalence of various microbial genera varies. Genera such as *Cladosporium*, *Halomonas*, and *Micrococcus* exhibited a widespread distribution, in fact, with the relative abundance of sequenced reads reaching 20% in at least 30% of the samples. In contrast, genera such as *Malassezia*, *Mucor*, and *Botrytis* displayed more variable distributions, with prevalence increasing slightly at intermediate abundance thresholds. Other genera, such as *Acinetobacter*, were less common or exhibited a less uniform distribution across the samples.

## 4. Conclusions

A key conclusion of this study is that a polyphasic approach, integrating both cultivable and metagenomic methods, provides a more comprehensive and insightful understanding of the microbiome under investigation. The results highlighted the value of combining these methodologies to capture the full diversity of the microbial community. Notable differences were observed between the culturable and metagenomic methods, underscoring the unique strengths and limitations of each approach. As expected, metagenomic analysis identified unculturable taxa in both bacterial and fungal communities that were not detected by culturable methods, likely due to limitations of growth media and laboratory conditions. Conversely, certain culturable taxa were absent from metagenomic data, potentially due to technical constraints such as DNA extraction efficiency or primer specificity. Through culturable methods, the dominant bacterial species identified were *R. pickettii* and *N. niigatensis*, while the most abundant fungal species included *A. pullulans*, *A. alternata*, and *C. allicinum*. In contrast, metagenomic analysis revealed *Halomonas* sp. and *Sphingomonas* sp. as the predominant bacterial taxa, with *Cladosporium* sp. emerging as the most abundant fungal genus. In general, *R. pickettii* was consistently found across all locations, confirming its role as a stable member of the grapevine endophytic community. The notable presence of *Halomonas* in both wild and domesticated grapevines, particularly in the wild Monte Fenera samples identified via the non-cultivable approach, may reflect historical soil conditions and marine influences from six million years ago. The dual use of ITS and LSU primers for fungal community identification proved valuable, with LSU primers offering enhanced genus-level resolution and enabling comparisons with culture-based methods. The recurrent presence of *A. alternata* and *C. allicinum* across various vineyard management systems supports the hypothesis that these species may contribute to grapevine health through potential biocontrol mechanisms.

Another key finding is that, despite some variations in microbial composition across different farming practices, statistical analyses revealed that vineyard management does not considerably impact the overall microbial community. This contrasts with previous studies that suggested agricultural practices could substantially alter microbial diversity. These findings suggest the presence of a relatively stable core microbiota in the analysed grapevine. This study is particularly significant as it represents the first integration and comparison of the *V. sylvestris* endophyte community obtained via metagenomic analysis with that of domesticated grapevines. However, further research is required to determine whether the observed resilience of the endophytic microbiota is due to its inherent adaptation to the grapevine environment, making it less susceptible to external influences.

Defining a stable endophytic community in grapevines remains complex, given the myriads factors that shape microbial composition. Cultivable techniques are crucial for examining the structure and functional mechanisms of these endophytes, particularly in identifying potential biocontrol agents for vineyards. These findings contribute to a deeper understanding of the intricate interactions between grapevines and their associated microbial communities, with important implications for vineyard management and the development of sustainable biocontrol strategies. The stability of certain endophytic species highlights their potential as biocontrol candidates, which could aid in reducing pesticide use in viticulture. Future research should focus on investigating the functional roles of these key microbial taxa in plant health and disease resistance, potentially offering innovative approaches to sustainable agriculture.

## Figures and Tables

**Figure 13:**
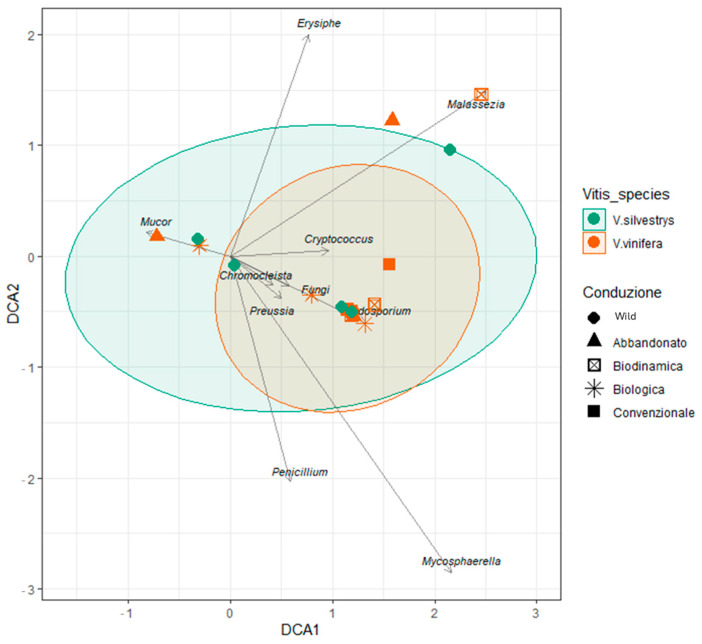
Detrended correspondence analysis (DCA). Points: represent samples, color-coded, and shaped according to the treatment condition treatments (abandoned, biodynamic, organic, and conventional). DCA1 and DCA2 represent the first and second principal components, respectively, capturing the majority of the variation in the microbial community composition across the samples.

**Table 1:** Grapevine’s information about species, conduction (management), cultivar/variety, and location. The ‘Lat/Long’ refers to the coordinates (latitudes and longitudes) of the vineyard. Approach: (c) cultivable, (nc) not cultivable.

Species	Conduction	Location	Cultivar	Lat/Long (°)	Approach
*V. vinifera*	Abandoned	Franciacorta	Chardonnay	45.5913902	c, nc
				10.1714237	
	Conventional (IPM)		Chardonnay	45.588739	c, nc
				9.934696	
	Conventional (IPM)		Chardonnay	45.61212	c
				10.01243	
	Biodynamic		Chardonnay	45.581510	c, nc
				10.016529	
	Organic		Chardonnay	45.656949	c, nc
				10.009343	
	Resistant to *P. viticola*	Riccagioia	Mgaloblishvili	44.982173	c
	Resistant to *P. viticola*		Kamuri Shavi	9.0917069	c
*V. vinifera* ssp. *sylvestris*	wild	Montalto	-	44.973866	c, nc
				9.2224803	
	wild	Monte Fenera	-	45.707653	c, nc
				8.310986	

**Table 2:** Primers and PCR conditions for DNA amplification. The ‘N’ column refers to the first PCR (amplification) and second PCR (adapter insertion) steps for library preparation. (Id) Initial denaturation, (D) denaturation, (A) annealing, (E) extension, (Cr) cycle repetition.

Region	N°	Primers	PCR Conditions
16S rRNA gene	First amplification	8F (5′-AGAGTTTGATCCTGG-3′)	Id: 98 °C for 30 s
			D: 98 °C for 10 s
			A: 56 °C for 30 s
		1492r (5′-GGTTACCTTGTTACG-3′)	E: 72 °C for 45 s
			Fe: 72 °C for 5 m
			Cr: 33 cycles
	Second amplification with adaptors	8F_ONT (5′-TTTCTGTTGGTGCTGATATTGCAGAGTTTGATCCTGGCTCAG-3′)	Id: 98 °C for 30 s
			D: 98 °C for 10 s
			A: 63 °C for 30 s
		1492r_ONT (5′-ACTTGCCTGTGCCTCTATCTTCGGTTACCTTGTTACGACTT-3′)	E: 72 °C for 45 s
			Fe: 72 °C for 5 m
			Cr: 20 cycles
ITS1-NL4 region	First amplification	ITSY1 (5′-TCGGTAGGTGAACCT-3′)	Id: 98 °C for 30 s,
			D: 98 °C for 10 s
			A: 54 °C for 1 m
		NL4 (5′-GGTCCGTGTTTCAAGACGG-3′)	E: 72 °C for 1 m
			Fe: 72 °C for 5 m
			Cr: 30 cycles
	Second amplification with adaptors	Ad-ITSY1 (5′-TTTCTGTTGGTGCTGATATTGCTCCGTAGGTGAACCTGCGG-3′)	Id: 98 °C for 30 s
			D: 98 °C for 10 s
			A: 68 °C for 1 m
		Ad-NL4 (5′-ATCTGCCTGTCGCTCTATCTTCGGTCCGTGTTTCAAGACGG-3′)	E: 72 °C for 1 m
			Fe: 72 °C for 5 m
			Cr: 25 cycles

## Data Availability

All data generated during this study are included in this article.

## Supplementary Materials

The following supporting information can be downloaded at: https://www.mdpi.com/article/10.3390/biology14030293/s1, Table S1: GenBank accession numbers of ITS sequences.; Table S2: GenBank accession numbers of 16S rRNA genes sequences.; Table S3: Metabarcoding GenBank accession numbers; Table S4: new generation of raw and filtered sequencing data; Table S5: influence of different agriculture practices on bacterial community—MANOVA statistical test; Figure S1: ordination methods to show the structure of bacterial communities associated with grapevine management and species.
