# Supplementary material for: Endophytic Diversity in Vitis vinifera with Different Vineyard Managements and Vitis sylvestris Populations from Northern Italy: A Comparative Study of Culture-Dependent and Amplicon Sequencing Methods
Source: Biology (Basel). 2025 Mar 14;14(3):293. doi: 10.3390/biology14030293 (PMC11940648; doi:10.3390/biology14030293)
Supplement: Supplementary file 1 [file biology-14-00293-s001.zip › Figure S1.pdf]

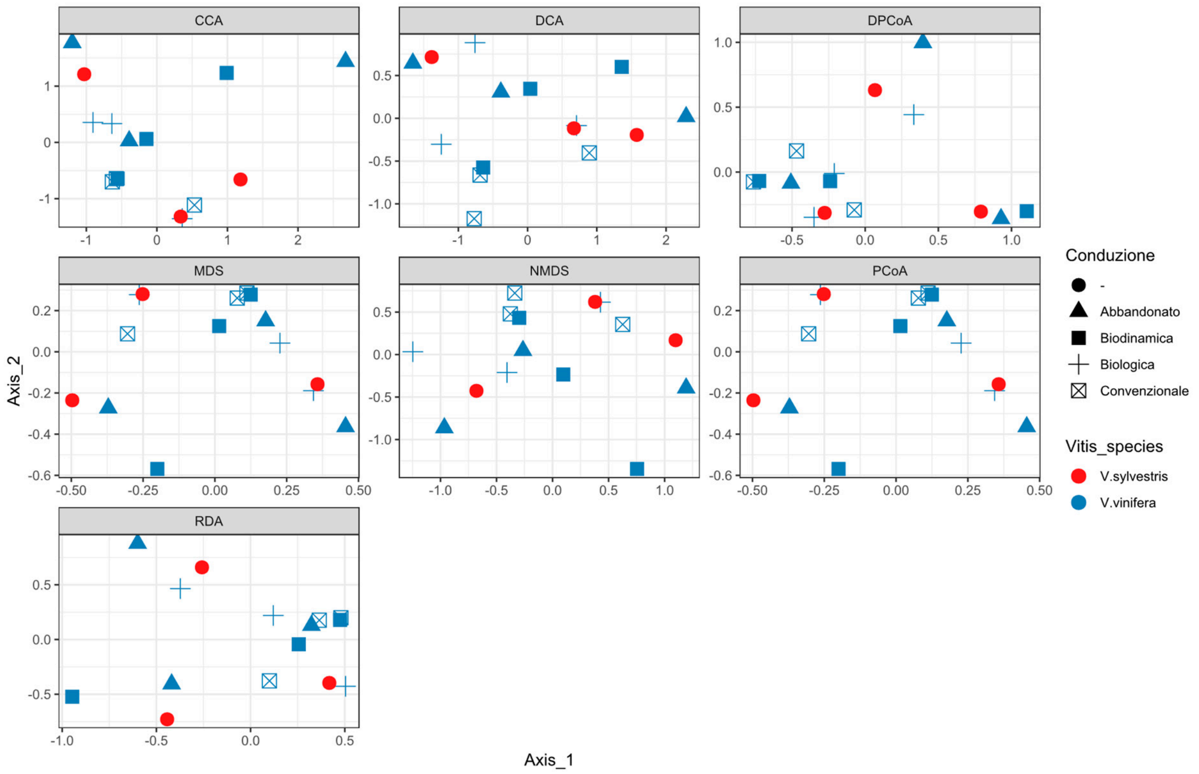

Figure S1. Ordination methods to show the structure of bacterial communities associated with grapevine management and species. CCA (Canonical Correspondence Analysis), DCA (Detrended Correspondence Analysis), DPCoA (Double Principal Coordinate Analysis), MDS (Multidimensional Scaling), NMDS (Non-metric Multidimensional Scaling), PCoA (Principal Coordinate Analysis), and RDA (Redundancy Analysis).
