# Supplementary material for: Endophytic Diversity in Vitis vinifera with Different Vineyard Managements and Vitis sylvestris Populations from Northern Italy: A Comparative Study of Culture-Dependent and Amplicon Sequencing Methods
Source: Biology (Basel). 2025 Mar 14;14(3):293. doi: 10.3390/biology14030293 (PMC11940648; doi:10.3390/biology14030293)
Supplement: Supplementary file 1 [file biology-14-00293-s001.zip › Table S1.pdf]

Table S1. GenBank accession numbers of ITS sequences.

**Fungal species**	**GenBank accession numbers**
Alternaria infectoria ED234	PQ580016
Aureobasidium pullulans ED73	PQ580017
Aureobasidium pullulans ED203	PQ580018
Aureobasidium pullulans ED206	PQ580019
Aureobasidium pullulans ED221	PQ580020
Chaetomium globosum ED85	PQ580021
Ciboria rufofusca ED83	PQ580022
Cladosporium allicinum ED79	PQ580023
Cryptovalsa ampelina ED200	PQ580024
Cytospora cedri ED94	PQ580025
Diatrype stigma ED197	PQ580026
Didymella pinodella ED174	PQ580027
Elsinoe salicina ED222	PQ580028
Filobasidium wieringae ED121	PQ580029
Paraconiothyrium brasiliense ED202	PQ580030
Plenodomus enteroleucus ED194	PQ580031
Talaromyces amestolkiae ED125	PQ580032
Alternaria alternata ED42	PQ580034
Alternaria alstroemeriae ED71	PQ580035
Diplodia seriata ED48	PQ580036
