# Supplementary material for: Endophytic Diversity in Vitis vinifera with Different Vineyard Managements and Vitis sylvestris Populations from Northern Italy: A Comparative Study of Culture-Dependent and Amplicon Sequencing Methods
Source: Biology (Basel). 2025 Mar 14;14(3):293. doi: 10.3390/biology14030293 (PMC11940648; doi:10.3390/biology14030293)
Supplement: Supplementary file 1 [file biology-14-00293-s001.zip › Table S2.pdf]

Table S2. GenBank accession numbers of 16S rRNA genes sequences.

**Bacterial species**	**GenBank accession numbers**
Bacillus megaterium ED84	PQ573130
Bacillus velezensis ED163	PQ573131
Bacillus cereus ED158	PQ573132
Brevibacillus parabrevis ED216	PQ573133
Curtobacterium flaccumfaciens ED52	PQ573134
Deinococcus citri ED140	PQ573135
Dermacoccus nishinomiyaensis ED226	PQ573136
Enterococcus faecium ED230	PQ573137
Kocuria rhizophila ED256	PQ573138
Massilia aurea ED53	PQ573139
Leifsonia shinshuensis ED105	PQ573140
Mycobacterium canariasense ED167	PQ573141
Mycobacteroides abscessus ED102	PQ573142
Mycolicibacterium aubagnense ED151	PQ573143
Nocardia niigatensis ED116	PQ573144
Okibacterium fritillariae ED58	PQ573145
Pantoea agglomerans ED77	PQ573146
Paracoccus yeei ED232	PQ573147
Pseudomonas coleopterorum ED74	PQ573148
Pseudomonas extremaustralis ED219	PQ573149
Ralstonia pickettii ED82	PQ573150
Staphylococcus warneri ED244	PQ573151
Sphingomonas echinoides ED81	PQ573152
