# Supplementary material for: Endophytic Diversity in Vitis vinifera with Different Vineyard Managements and Vitis sylvestris Populations from Northern Italy: A Comparative Study of Culture-Dependent and Amplicon Sequencing Methods
Source: Biology (Basel). 2025 Mar 14;14(3):293. doi: 10.3390/biology14030293 (PMC11940648; doi:10.3390/biology14030293)
Supplement: Supplementary file 1 [file biology-14-00293-s001.zip › Table S3.pdf]

Table S3. Metabarcoding GenBank accession numbers.

Sample title	GenBank accession number
1Lb	SAMN46343911
9Lb	SAMN46343912
17Lb	SAMN46343913
3Lf	SAMN46343914
11Lf	SAMN46343915
2Lb	SAMN46343916
10Lb	SAMN46343917
18Lb	SAMN46343918
4Lf	SAMN46343919
12Lf	SAMN46343920
3Lb	SAMN46343921
11Lb	SAMN46343922
19Lb	SAMN46343923
5Lf	SAMN46343924
14Lf	SAMN46343925
4Lb	SAMN46343926
12Lb	SAMN46343927
2Lb-CTAB	SAMN46343928
6Lf	SAMN46343929
15Lf	SAMN46343930
5Lb	SAMN46343931
13Lb	SAMN46343932
6Lb-CTAB	SAMN46343933
7Lf	SAMN46343934
16Lf	SAMN46343935
6Lb	SAMN46343936
14Lb	SAMN46343937
9Lb-CTAB	SAMN46343938
8Lf	SAMN46343939
17Lf	SAMN46343940
7Lb	SAMN46343941
15Lb	SAMN46343942
1Lf	SAMN46343943
9Lf	SAMN46343944
18Lf	SAMN46343945
8Lb	SAMN46343946
16Lb	SAMN46343947
2Lf	SAMN46343948
10Lf	SAMN46343949
19Lf	SAMN46343950
