# Supplementary material for: Endophytic Diversity in Vitis vinifera with Different Vineyard Managements and Vitis sylvestris Populations from Northern Italy: A Comparative Study of Culture-Dependent and Amplicon Sequencing Methods
Source: Biology (Basel). 2025 Mar 14;14(3):293. doi: 10.3390/biology14030293 (PMC11940648; doi:10.3390/biology14030293)
Supplement: Supplementary file 1 [file biology-14-00293-s001.zip › Table S4.pdf]

Table S4. New generation of raw and filtered sequencing data

Sample	Marker	TOT_reads (400<read<1800 bp)	TOT_Fail_reads (q<20)	TOT_Pass_reads (q>20)	Mapped_reads 16s	Mapped_ITS	Mapped_LSU
1Lb	16S	195625	57015	138610	90348		
9Lb	16S	607497	179600	427897	264246		
17Lb	16S	103961	33544	70417	44011		
3Lf	ITS-LSU_D1/D2	189151	45748	143403		142488	142488
11Lf	ITS-LSU_D1/D2	276493	66468	210025		182706	182706
2lb	16S	91488	29150	62338	36648		
10Lb	16S	124878	36681	88197	61263		
18lb	16S	128835	40162	88673	52938		
4Lf	ITS-LSU_D1/D2	315178	75059	240119		236881	236881
12Lf	ITS-LSU_D1/D2	82560	19230	63330		59094	59094
3Lb	16S	74108	21720	52388	37419		
11Lb	16S	102629	29903	72726	50427		
19Lb	16S	143400	34410	108990	74658		
5Lf	ITS-LSU_D1/D2	265661	63282	202379		201691	201691
14Lf	ITS-LSU_D1/D2	22420	3	22417		15985	15985
4Lb	16S	179452	50753	128699	92689		
12Lb	16S	136988	43315	93673	44871		
2Lb-CTAB	16S	143229	40875	102354	75528		
6Lf	ITS-LSU_D1/D2	22356	6514	15842		10851	10851
15Lf	ITS-LSU_D1/D2	114575	26821	87754		86312	86312
5Lb	16S	86241	24985	61256	43440		
13Lb	16S	93982	31644	62338	44362		
6Lb-CTAB	16S	173619	51211	122408	89355		
7Lf	ITS-LSU_D1/D2	22921	6532	16389		12194	12194
16Lf	ITS-LSU_D1/D2	85559	30651	54908		38303	38303
6Lb	16S	302374	89725	212649	136394		
14Lb	16S	93031	30945	62086	31589		
9Lb-CTAB	16S	74334	28102	46232	20340		
8Lf	ITS-LSU_D1/D2	175803	40757	135046		125292	125292
17Lf	ITS-LSU_D1/D2	88903	23976	64927		54076	54076
7Lb	16S	161649	47502	114147	80543		
15Lb	16S	168869	49408	119461	83836		
1Lf	ITS-LSU_D1/D2	21991	5664	16327		13875	13875
9Lf	ITS-LSU_D1/D2	30563	8367	22196		15651	15651
18Lf	ITS-LSU_D1/D2	23473	6591	16882		12479	12479
8Lb	16S	178827	30689	148138	52874		
16Lb	16S	84534	25191	59343	58705		
2Lf	ITS-LSU_D1/D2	54381	3696	50685		6768	6768
10Lf	ITS-LSU_D1/D2	83503	5603	77900		11028	11028
19Lf	ITS-LSU_D1/D2	20082	6328	13754		12147	12147
