# Supplementary material for: Endophytic Diversity in Vitis vinifera with Different Vineyard Managements and Vitis sylvestris Populations from Northern Italy: A Comparative Study of Culture-Dependent and Amplicon Sequencing Methods
Source: Biology (Basel). 2025 Mar 14;14(3):293. doi: 10.3390/biology14030293 (PMC11940648; doi:10.3390/biology14030293)
Supplement: Supplementary file 1 [file biology-14-00293-s001.zip › Table S5.pdf]

Table S5. Influence of different agriculture practices on bacterial community - MANOVA statistical test.

Groups of measurement	R2	p.value	p.adjusted	Significance
**Biologic vs Wild**	0.59	0.0892574992151741	0.712	0.712
**Biologic vs Abandoned**	0.736458898568306	0.128382145081195	0.615	0.683333333333333
**Conventional vs Wild**	103713845035916.00	0.147380708462007	0.433	0.54125
**Biodinamic vs Conventional**	103881923688878.00	0.147584303833883	0.393	0.54125
**Biologic vs Biodynamic**	107749685261807.00	0.152242646666736	0.316	0.535
**Abandoned vs Wild**	0.939677882190821	0.15820350881456	0.321	0.535
**Biologic vs Conventional**	127690700908848.00	0.175473866505878	0.19	0.535
**Biodynamic vs Wild**	13229279710632.00	0.180655603372148	0.296	0.535
**Biodynamic vs Abandoned**	144340405272917.00	0.224012655564849	0.208	0.535
**Conventional vs Abandoned**	16182107976956.00	0.244508802629715	0.125	0.535
